# Address of Dr. Taft before the Mississippi Valley Association of Dental Surgeons, February, 17, 1853

**Published:** 1853-04

**Authors:** 


### DENTAL REGISTER OF THE WEST.

Vol. VI. APRIL, 1853, No. 3.

### Original Communications.

### ADDRESS

*Of Dr. Taft before the Mississippi Valley Association of Dental Surgeons, February, 17, 1853.*

THE DENTAL PROFESSION—THE PAST AND FUTURE.

Gentlemen:—The work hitherto accomplished in the Dental Profession, occupies a field far too ample and extended, to examine, at this time, minutely; nor do I intend to refer to it, only as introductory to the main point of the present remarks.

Dental Science was, like other departments of medicine, developed by circumstances, whose nature was of an imperious character. It did not originate in the fancy, but had more urgent requirements to fulfill.

At first it did not enter the fields of science; but was content with an occasional glance in that direction; until the progressive *age* of the world began to dawn, when there was a "moving among the dry bones," and the mould and rust of ages began to be removed, and the arcanum of nature to be opened up; and a slight view given of her resources.

Here was a great field in which the mind of man could be exercised. The time had now arived when that lethargic mantle which pall-like had for so many ages enveloped the nations, must be removed. Instrumentalities to accomplish this spring into existence, with almost an incredible rapidity.

*Dental Science* was not developed at first with the rapidity of many other sciences; a very few years, even half a century will place it back in swadling cloths, and lower than in a manger. But at length the long extending arms of science took it up, and nourished it with more than a foster-mother's care.

But a few years ago the Dental profession possessed no weapons either of aggression or defence; nothing by which its claims could be sustained, or its cause advanced, save the individual efforts of a few devoted, ardent men, who espoused the dental profession, and labored assiduously for its elevation; planting their feet firmly upon the stepping stones of science, and drawing it with them or rather pushing it before them. Such men should ever be held in grateful remembrance, and, as the honored instruments, of its elevation.

Periodicals, Colleges, and Associations for the advancement of dental science are things of recent date.

In the present progressive age of the world, no cause can be rapidly advanced without the aid of the press; very significantly is it denominated the *lever* that moves the world; its arm is omnipotent. By the issue of its millions of pages daily, the radiant beams of light come "flashing o'er the land, and he who will not see must shut his eyes." The press is one agency by which dental science has been so rapidly developed. In this we see one prominent cause of the rapid, aye unparalleled progress of dental science in our own country; in which there are some eight or ten dental periodicals, when all the woild besides cannot boast of half so many.

The institutions—*our dental schools*, springing up in our land, all so many lights, from which beam forth a glorious radiance, illuminating the paths of those who desire the light; not only enlightening the paths of those who desire to walk therein, but casting forth a blaze of light, intolerable to those who would not be seen in their true character.

Our Colleges stand as the pillars that support the fair fabric of dental science. Dental Associations come in for a mead of glory in this work; a *great work* has indeed been accomplished by associations. Comparing methods of practice, and different theories; bringing new theories to the test, making them to pass under the *touch-stone* of truth.

It has been their prerogative, and properly too, to act in the capacity of an *umpire*, in many matters of a professional controversy. To elevate and sustain a standard of professional excellence.

We have now noted a *trio*—a noble *trio*—by whose combined agency dental science has been so rapidly developed in our own country; of the second and third of these our own country can boast the exclusive possession, and the greater part of the former. In other parts of the world it has been borne along by instrumentalities, whose aims and operations were complex; being esteemed, when treated with respect at all,—as one of the component departments of general medicine and surgery, always occupying the back ground even, of these; hence its tardy progress, *wherever* this course has been pursued.

Dental science has always most rapidly advanced, where it has been received and treated as a speciality, and *separate* department of medical science; and most tardy where the opposite policy has been observed. Yet, notwithstanding, these facts stand clearly before the eyes of the world, a few are found who *assume* or *possess* the ability to scan more closely the matter, than all others; such would, by action, if not by expression, set their foot upon the progressive spirit of the age, and arrest its onward course, were it in their power; but they are impotent, to stay, or even arrest the mighty tide.

By the agencies already referred to, the profession has been brought from a very low position, and made to occupy one corresponding with its true character.

Once, it was esteemed a mere trade or mechanical occupation. It now stands side by side with other honorable, scientific, and responsible professions.

Once, it was almost wholly, in the hands of *ignorance*, *charlatancy*, and *knavery*; now it can boast of men—in its foremost ranks—of *learning*, *science*, and *sterling integrity*.

There has been an elevation of all the departments of the profession. It was supposed, at one time, that mechanical skill and tact was all that was necessary to constitute a good dentist; yet, I doubt not, we have those in our midst to-day, whose memory extends to the time, when mechanical dentistry was only a matter of taste; when almost the entire amount of mechanical operations were matters of inconvenience and inutility. All are aware of the degree of perfection in this department at present.

Although this was at so low an ebb, the operative and more scientific departments, occupied a still more degraded position. It may well nigh be regarded an axiom, that the mechanical skill of the profession has always been in advance of the operative and more complex departments. This was the result of two considerations—the first true and the second false. Mechanical skill was more easily obtained, than a correct knowledge and practice of the more complex operations; and that the former was far more necessary than the latter.

There has been, not only great improvement in the mode of performing all the operations; but also, a corresponding improvement in the instruments and appliances by which such operations are executed.

Notwithstanding, much has already been accomplished, much yet remains to be done. Though we may not be competent to specify all that may or should be done, yet we can clearly see, that in many things, there are voids to be filled; there are modes to be changed; and much that will be altogether removed and otherwise occupied.

The supply of any commodity in the commercial world, is correspondent with the demand; the demand corresponds with the appreciation in which the commodity is held by community.

If society demands good dental operations, and properly appreciate them, then we will have skillful and scientific dentists.

But society cannot make a rational demand for that of which it is incompetent to judge.

We are then necessarily brought to the subject of *popular* instruction upon this subject.

The methods by which this may be accomplished, are numerous and varied.

The dentist can, in his office, give much valuable information to his patients, by clear and familiar conversation and illustration.

The public are anxious to gain information—prompted occasionally by vain curiosity, doubtless—but by far the greater part, by pure motives.

The professional man, who desires the elevation of his profession, and the welfare of his patients, will not fail upon all proper occasions, to give information in regard to his profession; it should be done in a quiet, unobtrusive, unassuming manner.

The quack seeks to cast the vail of mystery over every thing connected with his profession; this he does, that he may keep others in ignorance, that they may not "measure his shoal water;" but chiefly, that he may practice deception and fraud upon them.

There are other means of popular information, equally, if not more efficient; among which, we may notice the *publication* and *disrtribution* of popular treaties and essays upon dental science.

There are two extremes, here to be avoided; the one is a scientific research, description and illustration, too profound and abstruse for the general reader. The other is the issue of publications in such a manner, that they serve only as an advertising medium.

Whatever may be the ostensible design of the publications last referred to, the real design, is not popular instruction; they are generally characterized by two things: first, that there are a great many mysterious and wonderful things in dentistry; secondly, that the author has surmounted them all, can master all the difficulties, can perform all the wonderful operations, in the newest style, and most improved plan; or perhaps, upon some method, peculiarly his own—unknown to the whole world besides.

It could not be expected that each and every practitioner could or should write a popular treatise upon the whole routine of dental science; but that some small, well written, comprehensive work be obtained by each practitioner, for distribution in his practice. Each one might illustrate particular points in a popular manner, in the form of a few pages, or otherwise. Many could do this much, whose time would not permit them to prepare a complete treatise.

It is the duty of every dentist to write, it is a duty due to himself, to his profession, and to the community in which he lives. He who does not write, is recreant to the trust imposed upon him. Popular lectures from the various dental associations, would be efficient for good; they would, however, necessarily be quite limited in their influence, unless published for distribution.

Many other methods of popular information, not alluded to here, could profitably be adopted. The work must be done. If the dental profession is such, that the public should be kept in ignorance about it, the sooner it is abandoned the better.

When the public is properly informed, they will demand *perfect*, instead of *cheap* operations.

Much that we all ardently desire, will be accomplished when the community, or a considerable portion of it is properly informed, not upon all that pertains to dental science, but upon some of its fundamental principles.

The best means of preserving the natural teeth, is a subject in which all would take an interest, and this is the all important consideration with those who yet possess the natural organs in a state of perfection.

Those whose teeth have already been invaded by the ravages of disease, become particularly interested in the most perfect and efficient method of arresting such disease; while those who have lost the teeth, are especially interested about the best method of supplying the loss. While these are interests affecting directly, certain individuals of the public, yet all of the departments, indirectly, if not directly, should interest all of these individuals. In all of the departments of dentistry, is popular information necessary; not in the mechanical only—for as this is unduly exalted, so will the profession be esteemed a mere trade, and thus suffer degradation from its true position—but especially in the operative and more complex parts.

Let the opinions, entertained upon healthy and diseased conditions, upon surgical and mechanical operations, be based upon true and scientific ground.

Every practitioner of any observation, is fully aware of the errors, false opinions, prejudices, and rediculous theories entertained by the great body of the public, upon dental science and practice. To *remove* these, and substitute correct opinions and impressions in their stead, is a work to be accomplished *by the profession.*

Great proficiency is yet to be attained, in dental science; there will necessarily be much improvement, and greater perfection in the execution of *all* the dental operations now performed; however perfect they may now appear to us, and the declaration of many perfectionists—"that their operations are perfect, and not to be excelled," to the contrary, notwithstanding, there is much in every class of operations yet to be attained. All good operators know full well that their *best* operations are not *all* that they could desire, in every respect, it is here needless to specify.

Not only will there be much greater perfection attained in operations now performed, but each class of operations will be much extended. The present modes, however much improved, will not be the "*stand still*" point; new methods of operating, are continually being brought in practice.

There is yet much to be attained by our best operators—I speak with defference and respect—and very much more by those, upon whom time and experience, have not exercised their foster influence.

In the profession there must be a more exalted standard of general scientific knowledge; particularly those things which stand as collaterals to dental science; by this means it will be enabled to extend itself into all the ramifications of science,—select, and appropriate to its own development all materials susceptable of such appropriation.

The vast resources of the world, and especially of our own land are just beginning to be opened up to view. Noble men go forth, bearing in their hands the key of knowledge, and roll back the bars and bolts, that have for ages imprisoned nature's resources.

Our country contains as great variety, and richer profusion of materials for the development of science, than any country in the world. The natural abilities of the people of this country do not excel those of others; but circumstances have made greater acquired abilities; here any one who desires can enter the fields of science, and indulge to the extent of his capacity. In other countries it is not thus; forbidding circumstances so hedge in the great proportion of their people, that all the energies of body and intellect are exhausted in procuring a subsistance, and to this end they are directed.

The days of empiricism with its scathing, blighting, blasting influence, are gradually disappearing before the influences that are made to act against it; yet there is much to be done here, a great host to contend with, and but few to make battle. Yet considering the influences that are every day arising up against it, we prospectively see empiricism with all its ignorance, audacity, deceit, and dishonesty, laid low in the dust. Empiricism presents numerous aspects, but the mode of warfare need not be changed for every new phase.

The associations now in existence have a continually increasing work to perform. They are enabled by experience, to labor more efficiently than heretofore; they have the experience of years for reference. The good they have accomplished, is a scource of much gratification, interest and improvement. Wherein error has been committed, that, too, may *serve* as a beacon light to warn away from points of danger. Not only should the fields already occupied be cultivated, but new fields should be opened up.

New associations will be raised up. New schools opened, and periodicals multiplied. When all these agencies and others that will arise, shall have been brought, in their appropriate and extended influence to operate, then may we anticipate a progress and rapidity of development unparalleled in the annals of science.

